# Editorial: Human-centered solutions and synergies across robotic and digital systems for rehabilitation

**DOI:** 10.3389/frobt.2024.1462558

**Published:** 2024-10-29

**Authors:** Giacinto Barresi, Ana Lúcia Faria, Marta Matamala-Gomez, Edward Grant, Philippe S. Archambault, Giampaolo Brichetto, Thomas Platz

**Affiliations:** ^1^ Bristol Robotics Laboratory, University of the West of England, Bristol, United Kingdom; ^2^ Department of Psychology, Faculty of Arts and Humanities, University of Madeira, Funchal, Portugal; ^3^ NOVA Laboratory for Computer Science and Informatics, Caparica, Portugal; ^4^ Agência Regional para o Desenvolvimento da Investigação, Tecnologia e Inovação, Funchal, Portugal; ^5^ Department of Cognition, Development and Educational Psychology, University of Barcelona, Barcelona, Spain; ^6^ Cognition and Brain Plasticity Group, Bellvitge Biomedical Research Institute (IDIBELL), L’Hospitalet de Llobregat, Barcelona, Spain; ^7^ University School of Health and Sport (EUSES), University of Girona, Girona, Spain; ^8^ Department of Electrical and Computer Engineering, North Carolina State University, Raleigh, NC, United States; ^9^ School of Physical and Occupational Therapy, McGill University, Montreal, QC, Canada; ^10^ Centre for Interdisciplinary Research in Rehabilitation, Montreal, QC, Canada; ^11^ AISM Rehabilitation Center, Italian MS Society, Genoa, Italy; ^12^ BDH-Klinik Greifswald, Institute for Neurorehabilitation and Evidence-Based Practice, “An-Institut,” University of Greifswald, Greifswald, Germany; ^13^ Neurorehabilitation Research Group, University Medical Centre, Greifswald, Germany

**Keywords:** rehabilitation, robotics, artificial intelligence, digital health, extended reality, video games

The growing need for effective, personalized, clinically compliant, and engaging rehabilitation – based on methodologies for the progressive restoration of lost functions – can leverage the step-changes offered by interaction technologies to obtain optimal results matching the initial requests of the users (patients and clinicians). Human-Centered Design approaches may disclose the full potential of such solutions, especially considering the impact of smart systems powered by robotic devices and digital settings. In particular, virtual reality (VR) and augmented reality (AR) constitute a broad sub-class of digital settings, often intertwined with serious games (including exergames devised to promote training activities) and gamification (introducing game features in non-leisure solutions) for sustaining the users’ effort over time in repetitive exercises. Furthermore, they can be connected to smart mechatronic systems (especially through their artificial intelligence – AI – features) for achieving higher versatility and efficiency (making rehabilitation more sustainable for the individual and for the healthcare system as a whole, as in telerehabilitation frameworks) (Adlakha et al., 2020; Berton et al., 2020; Mohebbi, 2020; Shahmoradi et al., 2022).

Accordingly, this Research Topic aimed at collecting contributions on robotic and digital technologies for starting a wider dialectics on the groundbreaking opportunities offered by such innovations.

A first example of a digital system is proposed by Faria et al. in “*NeuroAIreh@b: an artificial intelligence-based methodology for personalized and adaptive neurorehabilitation*,” remarking on the contribution of AI on optimizing neuropsychological rehabilitation through a more objective cognitive profiling and a better personalization of cognitive training. Computer-powered rehabilitation solutions are presented by Barth et al., who promote the use of avatar-based game-like training in “*Functional improvement of patients with Parkinson syndromes using a rehabilitation training software*.”

Among the investigations focusing on digital solutions, “*Design recommendations for XR-based motor rehabilitation exergames at home*” is presented by Lorenz et al. to guide the design and development of novel Extended Reality (XR, the umbrella term for all types of combinations of virtuality and reality) settings for home training. Furthermore, “*A novel immersive virtual reality environment for the motor rehabilitation of stroke patients: A feasibility study*” by Fregna et al. remarks on the potential impact of this VR technology to improve the individual adherence to clinical protocols, one of the most crucial aspects to introduce immersive settings in healthcare. Innovative solutions for enriching the user experience in VR systems devised for clinical goals are discussed by Liu et al. in “*Augmented feedback modes during functional grasp training with an intelligent glove and virtual reality for persons with traumatic brain injury*.”

This Research Topic also explored studies based on mechatronic devices, such as in assistive or prosthetic robotic systems able to restore individual skills in activities of daily living (ADLs). In “*Use of an upright power wheelchair in spinal cord injury: a case series*,” Hong et al. discuss the advantages of using the mentioned device in terms of objective and subjective measures of the reactions of chronic, non-ambulatory people. Furthermore, Battraw et al. present the design and characterization of “*A multiarticulate pediatric prosthetic hand for clinical and research applications*,” highlighting its potential as a robust and accessible platform for translational investigations on bionic limbs.

About therapeutic interventions for motor recovery, Chambers and Artemiadis demonstrate how repeated unilateral stiffness perturbations might work for post-stroke gait re-training in their “*Using robot-assisted stiffness perturbations to evoke aftereffects useful to post-stroke gait rehabilitation*.” Robots can also work as mediators of exergames, as discussed by Fitter et al. in “*How should robots exercise with people? Robot-mediated exergames win with music, social analogues, and gameplay clarity*.” This last study, in particular, discloses the topic of hybrid solutions, the result of synergistic approaches mentioned in the title of this Research Topic. Indeed, the latter also aims at highlighting how synergies between digital and robotic systems can integrate and extend their advantages, offering higher versatility and engagement to their users. Such potential synergies are discussed by Albanese et al. through a SWOT (Strengths, Weaknesses, Opportunities, Threats) analysis of both robotic and virtual/augmented systems in “*Robotic systems for upper-limb rehabilitation in multiple sclerosis: a SWOT analysis and the synergies with virtual and augmented environments*,” proposing to adopt their synergies for powering each other.

Interestingly, examples of such synergies can be retrieved in papers focusing on humanoid systems. Indeed, Platz et al. presented their study on “*Feasibility, coverage, and inter-rater reliability of the assessment of therapeutic interaction by a humanoid robot providing arm rehabilitation to stroke survivors using the instrument THER-I-ACT*” with a focus on a digital-humanoid robotic platform for evidence-based upper limb rehabilitation (Platz et al., 2021). Here, the authors demonstrate that therapeutic interaction by a humanoid robot as social agent can comprehensively and reliably be coded in the same way as human therapists’ professional therapeutic interaction. In the paper “*Analysis of the therapeutic interaction provided by a humanoid robot serving stroke survivors as a therapeutic assistant for arm rehabilitation*” by Platz et al. it was documented that the digital therapy system E-BRAiN (Evidence-Based Robot Assistance in Neurorehabilitation; www.ebrain-science.de) that dynamically combines both knowledge about specific and diverse therapies (as implemented), therapeutic dialogue knowledge, and individual patient data showed therapeutic interaction (by the humanoid robot) that varied with type of therapy and over time (across therapeutic sessions) in as similar way as the interaction by human therapists providing the same types of therapy when administered to stroke survivors. Overall, these research papers remark on the opportunity of adopting anthropomorphic robots in combination with sophisticated digitalization of therapeutic guidance in clinical settings with a high degree of comparability to human therapy administration. This comprehensive comparability of humanoid robot-led therapy to therapy administration by human therapists opens a window of opportunity to integrate its use in healthcare settings, partially delegate tasks from human beings to humanoid robot-based systems, and hence to solve the pressing issue of an increasing demand for rehabilitation services globally and a shortage of healthcare workers globally (Feigin et al., 2023).

Summing up, this Research Topic presents several cases of investigations and solutions devised for employing the advantages of robotic and digital technologies (especially based on VR, AR, and XR paradigms) to enhance the clinical outcomes in rehabilitation, possibly extending their valuable contribution from laboratory tasks to ADLs. Possibly embracing the advantages of AI and neurotechnologies, these synergies between robotic and digital technologies pave the way for exploring novel ways to make rehabilitation systems truly centered on the human being, engaging people in repetitive activities (a task that XR systems and social robots can accomplish) and tailoring their specific clinical goals (as mechatronic devices can perform at physical level, and digital systems can in terms of cognition, behaviour, and social interaction). Intertwining the potential of both robotic and digital systems (individually and synergistically discussed in the examples provided by this Research Topic) can lead to versatile and impactful strategies in diverse types of rehabilitation: this is a perspective that should be included in the mindset of anyone working on the co-design of such technologies.
